# Community-Acquired Bacterial Meningitis in Alcoholic Patients

**DOI:** 10.1371/journal.pone.0009102

**Published:** 2010-02-08

**Authors:** Martijn Weisfelt, Jan de Gans, Arie van der Ende, Diederik van de Beek

**Affiliations:** 1 Department of Neurology, Kennemer Gasthuis, Haarlem, The Netherlands; 2 Netherlands Reference Laboratory for Bacterial Meningitis, Department of Neurology, Center for Infection and Immunity Amsterdam (CINIMA), Academic Medical Center, Amsterdam, The Netherlands; 3 Netherlands Reference Laboratory for Bacterial Meningitis, Department of Microbiology, Center for Infection and Immunity Amsterdam (CINIMA), Academic Medical Center, Amsterdam, The Netherlands; University of California Merced, United States of America

## Abstract

**Background:**

Alcoholism is associated with susceptibility to infectious disease, particularly bacterial pneumonia. In the present study we described characteristics in alcoholic patients with bacterial meningitis and delineate the differences with findings in non-alcoholic adults with bacterial meningitis.

**Methods/Principal Findings:**

This was a prospective nationwide observational cohort study including patients aged >16 years who had bacterial meningitis confirmed by culture of cerebrospinal fluid (696 episodes of bacterial meningitis occurring in 671 patients). Alcoholism was present in 27 of 686 recorded episodes of bacterial meningitis (4%) and alcoholics were more often male than non-alcoholics (82% vs 48%, *P* = 0.001). A higher proportion of alcoholics had underlying pneumonia (41% vs 11% *P*<0.001). Alcoholics were more likely to have meningitis due to infection with *Streptococcus pneumoniae* (70% vs 50%, *P* = 0.01) and *Listeria monocytogenes* (19% vs 4%, *P* = 0.005), whereas *Neisseria meningitidis* was more common in non-alcoholic patients (39% vs 4%, *P* = 0.01). A large proportion of alcoholics developed complications during clinical course (82% vs 62%, as compared with non-alcoholics; *P* = 0.04), often cardiorespiratory failure (52% vs 28%, as compared with non-alcoholics; *P* = 0.01). Alcoholic patients were at risk for unfavourable outcome (67% vs 33%, as compared with non-alcoholics; *P*<0.001).

**Conclusions/Significance:**

Alcoholic patients are at high risk for complications resulting in high morbidity and mortality. They are especially at risk for cardiorespiratory failure due to underlying pneumonia, and therefore, aggressive supportive care may be crucial in the treatment of these patients.

## Introduction

Alcohol is the most commonly abused substance in the Western world is associated with impaired general health [1], [2]. Alcoholism has been clearly linked to increased host susceptibility to infectious disease, particularly bacterial pneumonia, probably due to decreased innate and adaptive immunity [1], [2]. We previously described clinical features and prognostic factors in 696 episodes of community-acquired bacterial meningitis in adults [3]. In the present analysis, we describe characteristics of alcoholic patients with bacterial meningitis and delineate differences with non-alcoholic with bacterial meningitis.

## Methods

### Participants and Investigations

The Dutch Meningitis Cohort Study, a prospective nationwide observational cohort study in the Netherlands, included 696 episodes of community-acquired acute bacterial meningitis in adults. Inclusion and exclusion criteria are described more extensively elsewhere [3]. In summary, eligible patients were aged >16 years who had bacterial meningitis confirmed by culture of cerebrospinal fluid (CSF) and were listed in the database of the Netherlands Reference Laboratory for Bacterial Meningitis from October 1998 to April 2002. This laboratory receives CSF isolates from approximately 85 percent of all patients with bacterial meningitis in the Netherlands [3], [4]. The laboratory provided daily updates of the names of hospitals where patients with bacterial meningitis had been admitted 2–6 days previously. The start of the cohort study was announced in the journal of the Dutch Neurological Society, with periodic reminders. Before the study started, all neurologists received by mail information about the study, including a case record form. The treating physician was contacted and was requested to ask the patient or their legally representatives for consent.

Information was obtained with a case record form. Despite the low median percentage of missing values for individual variables (2 percent), only 320 of the 696 patients had complete data on all predictors. In the current study, we dichotomized the cohort with respect to alcoholism. Alcohol dependence or alcoholism was defined according to the diagnostic criteria of the National Institute on Alcohol Abuse and Alcoholism (NIAAA), as a persistent and progressive pattern of abnormally intense alcohol-seeking behaviour that, over time, results in: loss of control over drinking, a preoccupation with drinking, compulsion to drink/unable to stop and the development of tolerance and dependence [5]. The interpretation of these criteria was left to the discretion of the treating physician. Strict quantitative criteria for the diagnosis of alcoholism (e.g., number of alcoholics consumptions per day) were lacking. Patients using immunosuppressive drugs and those with diabetes mellitus, alcoholism, asplenia, or HIV infection were considered to be immunocompromised. Focal neurologic abnormalities were categorized into focal cerebral deficits (aphasia, mono- or hemiparesis) and cranial nerve palsies. Complications during clinical course were divided into systemic complications (cardiorespiratory failure and sepsis). Brain infarction on CT was defined as focal hypodense area, in cortical, subcortical, or deep gray or white matter, following vascular territory, or watershed distribution. Early subtle findings include obscuration of gray/white matter contrast and effacement of sulci, or “insular ribbon” [6]. In the present study we distinguished cerebritis and brain abscess. The early stage of abscess formation was termed cerebritis, a pathologic finding [7]. The progression to encapsulation and abscess formation is a continuum. In cerebritis the precontrast CT only reveals an irregular poorly circumscribed area of low density. Post contrast CT shows ring enhancement with a variable rim of enhancement, which may be smooth or “flare out” into the brain with smooth to variable inner margins. Diffusion of contrast material into the central lucency is characteristic of early cerebritis. This central diffusion becomes less prominent in the late cerebritis stage and ceases with the appearance of encapsulation [7]. A neurologist performed a neurologic examination at discharge and outcome was graded by means of the Glasgow Outcome Scale (GOS). This is a well validated measurement scale with scores varying from 1 (indicating death) to 5 (good recovery) [8]. A favourable outcome was defined as a score of 5, and an unfavourable outcome as a score 1–4 [3].

To estimate the impact of complications, we categorized the cause of death in patients that died within 14 days after admission, as death within this period is likely to be caused by direct consequences of the meningitis [9]–[11]. Two experienced clinicians (MW, DvdB) independently classified the cause of death into systemic causes (e.g., septic shock, respiratory failure, multiple-organ dysfunction, cardiac ischemia) or neurologic causes (e.g., brain herniation, cerebrovascular complications, intractable seizures and withdrawal of care because of poor neurologic prognosis). We assessed interrater agreement by calculation of the kappa coefficient and differences in categorization between both clinicians were resolved by discussion [10], [11].

Penicillin susceptibility of meningococci and pneumococci was determined as described previously [12]. The microbial coverage of the empirical antibiotic therapy (defined as the antibiotic regimen started on admission in the hospital) was categorized as adequate or inadequate coverage. The microbial coverage of antibiotic therapy for *Neisseria meningitidis, Streptococcus pneumoniae* and *Haemophilus influenzae* was based on the results of *in vitro* antibiotic susceptibility testing. Intermediate resistance for penicillin was categorized as inadequate coverage if penicillin monotherapy was given. For other isolates coverage was categorized by an experienced microbiologist and was based on the antimicrobial spectrum of the antibiotic agents [13].

### Ethics

Written informed consent was obtained from all participating patients or their legally authorised representatives. The Dutch Meningitis Cohort Study was approved by the ethics committee of the Academic Medical Center in Amsterdam.

### Statistical Methods

Descriptive results of continuous data are expressed as medians and interquartile range. To identify differences between groups the Mann–Whitney *U*, Kruskal–Wallis, χ^2^, or Fisher's exact statistics were used. Analyses were carried out with SPSS, version 16.0.

## Results

The study included a total of 696 episodes of bacterial meningitis occurring in 671 patients; 25 patients had a second episode during the study period. Alcoholism was present in 27 of 686 recorded episodes of bacterial meningitis (4%), occurring in 27 patients. Several differences were found between the patients' characteristics on admission between alcoholic and non-alcoholic patients (Table 1). Patients were male in a higher proportion of alcoholic patients (22 of 27 [82%] vs 319 of 659 [48%], *P* = 0.001) and underlying pneumonia was more often present in alcoholic patients (11 of 27 [41%] vs 69 of 659 [11%], *P*<0.001). The rate of patients with an immunocompromised state was higher in alcoholic patients (100% vs 13%, *P*<0.001); alcoholism was one of the predefined criteria for immunocompromise. If alcoholism was excluded as one of the predefined criteria the presence of immunocompromise was similar in both groups (3 of 27 [11%] vs 86 of 659 [13%], *P* = 0.77). Clinical characteristics on presentation were similar between alcoholic and non-alcoholic patients, although non-alcoholic patients were more likely to have a rash than alcoholic patients (174 of 646 [27%] vs 1 of 27 [4%], *P* = 0.006).

**Table 1:** Patient's characteristics and clinical features in alcoholic and non-alcoholic patients with bacterial meningitis. a

*Characteristics*	*Alcoholism (n = 27)*	*Non-alcoholism (n = 659)*	*p-Value*
Patients' characteristics before admission			
Age	53 (45–64)	51 (32–67)	P = 0.52
Male gender	22 (82)	319 (48)	P = 0.001
Duration of symptoms <24 hours	7/23 (30)	305/630 (48)	P = 0.14
Seizures	3/23 (13)	29/635 (5)	P = 0.10
Headache	3/17 (18)	78/602 (13)	P = 0.48
Antibiotics before admission	4 (15)	57/656 (9)	P = 0.29
Predisposing factors b	27 (100)	282 (43)	P < 0.001
Otitis/sinusitis	3 (11)	172 (26)	P = 0.11
Pneumonie	11 (41)	69 (11)	P<0.001
Immunocompromise c	27 (100)	86 (13)	P<0.001
Clinical characteristics on presentation			
Neck stiffness	19/25 (76)	541/650 (83)	P = 0.41
Heart rate >120 beats per minute	8/24 (30)	69/619 (11)	P = 0.004
Systolic blood pressure	150 (121–183)	140 (120–160)	P = 0.14
Diastolic blood pressure	90 (74–96)	80 (65–90)	P = 0.04
Body temperature ≥38°C	19/25 (76)	493/644 (77)	P = 1.00
Rash	1 (4)	174/646 (27)	P = 0.01
Score on Glasgow Coma Scale d	11 (9–13)	11 (9–14)	P = 0.19
Classic triad of bacterial meningitis e	14 (52)	284 (43)	P = 0.37
Focal neurologic deficits f	11 (41)	217 (33)	P = 0.40
Focal cerebral deficits g	8 (30)	146 (22)	P = 0.36
Cranial nerve palsies	10 (37)	179 (27)	P = 0.26

a Data are number/number evaluated (%) or median (interquartile range);

b Defined as otitis/sinusitis, pneumonia or immunocompromise;

c defined as the use of immunosuppressive drugs or the presence of diabetes mellitus, alcoholism, asplenia, or HIV infection;

d scores on the Glasgow Coma Scale range from 3 to 15, with 15 indicating a normal level of consciousness;

e defined as fever, neck stiffness, and change in mental status;

f defined as the presence of focal cerebral deficits or cranial nerve palsies;

g defined as aphasia, mono- or hemiparesis.

Lumbar puncture was performed in all patients. CSF culture in the 27 alcoholic patients yielded: *S. pneumoniae* in 19 episodes (70%), *L. monocytogenes* in 5 (19%), *N. meningitidis* in 1 (4%), group B streptococcus in 1 (4%) and *Staphylococcus aureus* in 1 (4%; Table 2). Whereas alcoholic patients were more likely to have meningitis due to infection with *S. pneumonia* (70% vs 50%; p = 0.01) and *L. monocytogenes* (19% vs 4%; *P* = 0.005), *N. meningitidis* was much more common in non-alcoholic patients (39% vs 4%; *P* = 0.01). A CSF leukocyte count below 1000 cells/mm^3^ occurred more often in alcoholic patients (17 of 26 recorded episodes [65%] vs 162 of 609 [27%], *P*<0.001). Other indexes of inflammation in CSF (CSF protein level and CSF:blood glucose ratio) as well in the blood were similar in both groups.

**Table 2:** Laboratory results in alcoholic and non-alcoholic patients with bacterial meningitis. a

*Characteristics*	*Alcoholism (n = 27)*	*Non-alcoholism (n = 659)*	*p-Value*
Indexes of inflammation in CSF			
White-cell count per mm^3^	549 (141–2677)	3157 (880–8733)	P <0.001
<100	6/26 (23)	56/609 (9)	P <0.001
100–999	11/26 (42)	106/609 (17)	P <0.001
1000–9999	7/26 (27)	315/609 (52)	P <0.001
>10.000	2/26 (8)	132/609 (22)	P <0.001
Protein, g/L	4.2 (1.77–5.45)	4.24 (2.36–6.90)	P = 0.46
CSF:blood glucose ratio	0.058 (0.02–0.26)	0.07 (0.01–0.28)	P = 0.89
Indexes of inflammation in blood			
ESR, mm/hour	50 (41–77)	38 (18–69)	P = 0.06
CRP	240 (153–360)	214 (127–312)	P = 0.46
Sodium, mmol/L	135 (131–137)	137 (134–139)	P = 0.16
Glucose, mmol/L	10.8 (6.8–12.5)	9.0 (7.4–11.0)	P = 0.24
Thrombocyte count (×10^9^/L)	162 (98–239)	185 (142–240)	P = 0.12
Blood culture	16/20 (80)	381/582 (66)	P = 0.18
Gram stain			
Gram positive cocci	19/25 (76)	300/619 (48)	P = 0.01
Gram negative cocci	1/25 (4)	218/619 (35)	P = 0.001
Other bacteria	0	21/619 (3)	P = 0.35
Negative	5/25 (20)	80/619 (13)	P = 0.31
CSF culture			
*Streptococcus pneumoniae*	19/27 (70)	326/659 (49)	P = 0.01
*Neisseria menningitidis*	1/27 (4)	255/659 (39)	P = 0.01
*Listeria monocytogenes*	5/27 (19)	25/659 (4)	P = 0.01
Other bacteria	2/27 (7)	53/659 (8)	P = 0.91

a Data are number/number evaluated (%) or median (interquartile range);

CSF: cerebrospinal fluid; ESR: erythrocyte sedimentation rate; CRP: C-reactive protein.

Cranial CT was done for 19 (70%) episodes in alcoholic patients (Table 3). Abnormal findings were associated with 8 of these episodes (42%): brain infarction and cerebritis were the most common abnormalities, and 4 episodes were associated with more than one abnormality. In only 2 of 19 (11%) episodes, CT provided information about potential underlying disorders for meningitis (otitis or sinusitis or post-traumatic abnormalities). Intracranial abnormalities were deemed to be caused by the meningitis (recent brain infarction, brain swelling, hydrocephalus, empyema) for 8 (42%) episodes. A chest radiograph was done for 24 (89%) episodes and showed findings indicative for pneumonia for 11 (46%). An additional sinus radiograph was undertaken in only one patient and revealed no abnormalities.

**Table 3:** Results of cranial computed tomography in 27 alcoholic patients with bacterial meningitis.

*CT Findings*	*On admission (n = 14) no (%)*	*Admission and clinical course (n = 19) no (%)*
Total number of abnormalities a,b	6 (43)	8 (42)
Recent brain infarction	1 (7)	3 (16)
Sinusitis/otitis	1 (7)	1 (5)
Cerebral oedema	1 (7)	1 (5)
Hydrocephalus	1 (7)	0
Cerebritis	1 (7)	3 (16)
Empyema/abscess	0	1 (5)
Skull fracture	1 (7)	1 (5)

a Percentages are calculated per number of episodes with cranial CT performed;

b numbers do not add up to totals because of the presence of multiple abnormalities in several patients;

CT: computed tomography.

The empirical antibiotic regimen was recorded in 26 of alcoholic patients (96%). Antimicrobial treatment consisted of penicillin or amoxicillin in 12 (46%), the combination of third-generation cephalosporins with penicillin or amoxicillin in 7 (27%), the combination penicillin or amoxicillin with another antibiotic in 3 (12%) and the combination of third-generation cephalosporins with penicillin or amoxicillin with another antibiotic in 3 (12%). Microbial coverage of antibiotic therapy was categorized as adequate in all 26 evaluated episodes. Only 2 of the alcoholic patients (7%) received treatment with adjunctive steroids.

Complications were significant more likely to develop in alcoholics than in non-alcoholic patients (22 [81%] vs 408 [62%], *P* = 0.04; Table 4). Alcoholics and non-alcoholics developed neurologic complications in a similar proportion of patients (67% vs 53%). Systemic complications occurred in a higher proportion of alcoholics as compared to non-alcoholic patients (14 [52%] vs 187 of 659 [28%], *P* = 0.01). The results of a multivariate analysis for prognostic factors in our cohort, which included 21 potentially relevant determinants of outcome, has been described extensively elsewhere [3] Although in this analysis an immunocompromised state tended toward statistic significance, the presence of alcoholism was not identified as an independent risk factor for adverse outcome.

**Table 4:** Clinical course and outcome in alcoholic and non-alcoholic patients with bacterial meningitis.

*Characteristics*	*Alcoholism (n = 27) no (%)*	*Non-alcoholism (n = 659) no (%)*	*p-Value*
Clinical course			
Systemic complications a	14 (52)	187 (28)	P = 0.01
Cardiorespiratory failure	14 (52)	183 (28)	P = 0.01
Mechanical ventilation	13/14 (93)	143/181 (79)	P = 0.31
Sepsis b	8/18 (44)	80/376 (21)	P = 0.04
Hyponatremia c	11/26 (42)	172/581 (28)	P = 0.12
Neurologic complications d	18 (67)	348 (53)	P = 0.39
Focal neurologic deficits e	7 (26)	216 (33)	P = 0.46
Seizures	7 (26)	99/650 (15)	P = 0.17
Impairment of consciousness	14 (52)	256 (39)	P = 0.18
Scores on Glasgow Outcome Scale			
1 (death)	9 (33)	131 (20)	P = 0.09
2 (vegetative state)	0	3 (1)	P = 0.73
3 (severe disability)	4 (15)	20 (3)	P = 0.001
4 (moderate disability)	5 (19)	62 (9)	P = 0.76
5 (mild or no disability)	9 (33)	443 (66)	P = 0.001
Neurologic examination at discharge			
Focal neurologic deficits e	11/27 (41)	202/659 (31)	P = 0.27
Cranial-nerve palsies	3/16 (19)	102/460 (22)	P = 0.08
Focal cerebral deficits f	3/18 (17)	42/517 (8)	P = 0.19

a Defined as cardiorespiratoiy failure, need for mechanical ventilation or the presence of sepsis;

b defined as systolic blood pressure <90 mmHg with positive blood culture;

c defined as sodium<130 mmol per liter;

d defined as impairment of consciousness, seizures and focal neurologic abnormalities;

e defined as the presence of focal cerebral deficits or cranial nerve palsies;

f defined as aphasia, mono- or hemiparesis.

In total 140 of 686 patients (20%) died during hospitalization. Although the mortality rate in alcoholic patients did not differ significantly with that in non-alcoholic patients (9 [33%] vs 131 [20%]), alcoholic patients were more likely to have an unfavourable outcome (18 patients [67%] vs 216 patients [33%], *P*<0.001). Among the 9 alcoholic patients that died the causative pathogens were *S. pneumoniae* in 7 (78%), group B Streptococcus in 1 (11%) and *Staphylococcus aureus* in 1 (11%). A total of 117 of 140 (84%) patients died within two weeks after admission. Six alcoholic patients died within two week after admission and death was attributed to systemic complications in 3 patients and to neurologic complications in the remaining 3 patients. These rates were similar to those in non-alcoholic patients in which 65 of 111 (59%) patients died due to systemic causes and 46 (41%) due to neurological causes.

## Discussion

Our study shows that bacterial meningitis in alcoholic patients is associated with a very high rate of unfavourable outcome (67%). The most common causative pathogen among alcoholic patients was *S. pneumoniae* (70%). Infection with *S. pneumoniae* has been identified as an important risk factor for unfavourable outcome in many previous studies, but never such a high rate of unfavourable outcome [3], [14]. The severity of bacterial meningitis in alcoholic patients was also reflected by the high rate of cardiorespiratory failure often resulting in mechanical ventilation. This finding is in line with the high proportion of patients with underlying pneumonia present (46%) and implies that optimal supportive care may be crucial in the treatment of alcoholic patients with bacterial meningitis.

Patient's characteristics and clinical features on presentation were highly similar in alcoholic and non-alcoholic patients. Alcoholics were less likely to have a rash than non-alcoholic patients, which can be explained by the low rate (4%) of patients with *N. meningitidis* meningitis. Alcoholic patients were more likely to have a CSF leukocyte count below 1000 cells/mm^3^. Other indexes of inflammation in blood and CSF were highly similar in alcoholics and non-alcoholic patients. Leukocyte recruitment is a key aspect of the host response against invading micro-organisms and the weak leukocyte response in the CSF in alcoholic patients therefore may suggest an impaired host defence infection.

*S. pneumoniae* and *L. monocytogenes* were the most common causative bacteria of meningitis in alcoholic patients. Empirical treatment should be based on the most common bacterial species that cause the disease in the different patients groups depending on age, the presence of risk factors and the clinical setting, as well as on local antibiotic susceptibility patterns of the predominant pathogens [14]–[16]. In the Netherlands, the antibiotic susceptibility patterns of the predominant pathogens in bacterial meningitis are reported annually, and the rate of penicillin-resistance is low (<1%) [3], [16]–[18]. In the Dutch national guidelines for bacterial meningitis, a combination of penicillin or amoxicillin with a third-generation cephalosporin is recommended as empirical antibiotic therapy in alcoholics, patients aged over 60 years or patients with other risk factors present (e.g., diabetes mellitus, immunodeficiency or CSF leakage) [16]–[18]. In the present study only 37 percent of the physicians adhered to the recommendations contained within the guidelines for empirical antimicrobial therapy for patients with a history of alcohol abuse. Such low compliance rates have been reported before [12].

Alcoholism was associated with a high rate of complications during clinical course. Substantial clinical evidence suggests that alcohol abuse suppresses both innate and adaptive immune responses leading to an increased risk for infections and cancer, and delayed recovery from trauma [19]. Malnutrition and/or malabsorption are almost invariably associated with chronic alcohol abuse and are an important contributor to immunosuppresion and increased susceptibility to infections [20]. In a previous prospective study which included 1505 patients admitted to a general surgical department heavy alcohol consumption was associated with an increased risk of nosocomial infection in men who underwent general surgical procedures [21]. The fact that alcoholism was not identified as an independent risk factor for adverse outcome may be related to the strong collinearity between alcoholism and *S. pneumoniae* meningitis in our cohort.

### Limitations

This study has several limitations. The most important limitation of our study is the lack of strict quantitative criteria for the diagnosis of alcoholism. In the present study alcoholism was defined according to the diagnostic criteria of the National Institute on Alcohol Abuse and Alcoholism (NIAAA) alcohol dependence or alcoholism [5]. Therefore, it was not exactly noted if patients were current or formerly at risk drinkers or whether alcohol-induced physical harm, such as liver impairment due to fibrosis, was present. While the diagnostic criteria for alcohol dependence and alcohol abuse provided in current diagnostic schemes have contributed to improved case recognition, research has begun to focus on developing quantitative representations of these criteria using statistical methods that provide differential severity weighting for individual symptoms of alcoholism [5]. The development of quantitative criteria will lead to better understanding of the pathological stages of the disease, provide researchers an improved understanding of the aetiology of alcohol dependence, and facilitate categorization and severity determination in individual patients [5]. The proportion of patients with alcoholism present (4%) in our study was in line with a previous study that estimated that of adults from Western countries aged 18–59 year, 4.6% admitted to medical and surgical wards of hospitals have an alcohol use disorder [22], [23]. A recent prospective cohort study in non-abstaining individuals (mean age 42.9 years at study inclusion) from the UK-wide Health and Lifestyle Survey revealed alcohol problems in 2.4% of women and 7.8% of the male participants [24]. Therefore, the rate of alcoholism is conform expectations.

Questions about typical quantities of alcohol consumed often lead to underestimates. Therefore, it can be hypothesized that determining drinking patterns primarily based on information from patients and their family members may have resulted in a underestimation of the proportion of patients with alcoholism [25]. Although bacterial infections are frequent in patients with liver cirrhosis, case series of bacterial meningitis in alcoholic patients are scarce. A previous retrospective French study identified 16 cases of bacterial meningitis in patients with cirrhosis, of which thirteen had alcoholic cirrhosis [26]. The CSF culture was positive in 14 (88%) patients and revealed Gram-negative bacilli (mainly *Escherichia coli*) and *L. monocytogenes* in the majority of cases (64%). In contrast, *S. pneumoniae* and *N. meningitidis* were found in only 29% of the cases. It can be hypothesized that the discrepant high proportion of patients with *S. pneumoniae* meningitis indicates our study mainly included patients with less severe drinking problems without organ failure complications such as alcoholic cirrhosis. Despite this limitation, this study is the most comprehensive nationwide cohort study on alcoholism in bacterial meningitis to date.

The present study only included patients who had a positive CSF culture. Negative CSF cultures are estimated to occur in 11 to 30 percent of patients with bacterial meningitis [3], [14], [27]. However, as the clinical presentation in patients with positive and negative CSF cultures was similar in several studies this is unlikely to have significantly biased our results [3], [14], [27]. All patients in this study underwent lumbar puncture. In patients with septic shock or space-occupying lesions on CT lumbar puncture is generally not performed or postponed [3], [27], [28]. Therefore, these groups of patients were probably only partly included in our study. This may have resulted in selection bias and underestimation of the mortality rate. Finally, most patients in our study did not receive treatment with adjunctive steroids. In a recent placebo-controlled trial for adjunctive dexamethasone therapy in adults with bacterial meningitis, treatment with dexamethasone was associated with a reduction in the risk of an unfavourable outcome (relative risk, 0.6; 95% CI, 0.3–0.9; *P* = 0.03) and mortality (relative risk of death, 0.48; 95% CI, 0.2–0.96; *P* = 0.04) [29]. The beneficial effect was most striking in adults with pneumococcal meningitis, in whom mortality was reduced from 34 to 14 percent [29]. A subsequent meta-analysis showed that adjunctive steroid therapy also reduced neurologic sequelae among surviving patients [30]. As adjunctive dexamethasone has become routine therapy in most adults with bacterial meningitis[31], this may affect the generalizability of our results.

In conclusion, our study shows that bacterial meningitis in alcoholic patients is a disease with high incidence of complications, which results in high morbidity and mortality rates. Alcoholic patients develop complications in a high proportion of patients, which often consists of cardiorespiratory failure due to underlying pneumonia. Therefore, aggressive supportive care may be crucial in the treatment of alcoholic patients with bacterial meningitis.
